# Supplementary material for: Associations of Diet Quality and Sleep Quality with Obesity
Source: Nutrients. 2021 Sep 13;13(9):3181. doi: 10.3390/nu13093181 (PMC8466020; doi:10.3390/nu13093181)
Supplement: Supplementary file 1 [file nutrients-13-03181-s001.zip › nutrients-1364806-supplementary.pdf]

Supplementary file

Table S1. Correlation between Recommended Food Score (RFS) and serum carotenoid concentrations.

	Men (*n*=737)		Women (*n*=428)	
	**Correlation coefficient**	***p*-Value ***	**Correlation coefficient**	***p*-Value ***
Total carotenoids (μM)	0.081	0.027	0.135	0.005
Lutein (μM)	0.076	0.040	0.110	0.023
Zeaxanthin (μM)	0.010	0.781	0.067	0.167
ß-cryptoxanthin (μM)	0.037	0.313	0.104	0.031
α-carotene (μM)	0.056	0.130	0.135	0.005
ß-carotene (μM)	0.106	0.004	0.139	0.004

* Correlation coefficient and *p*-values were obtained by Pearson's correlation analysis.

Table S2. Blood pressure and blood profiles according to Recommended Food Score (RFS).

	Men					Women					*p*-Value ‡
	Total (N=737)	RFS ≤ median† (N=377)	RFS > median† (N=360)	*p*-Value *	*p*-Value **	Total (N=428)	RFS ≤ median† (N=220)	RFS > median† (N=208)	*p*-Value *	*p*-Value **	
SBP (mmHg)	123.6 ±12.6	123.3 ± 12.8	123.9 ± 12.3	0.524	0.584	110.8 ± 13.2	110.1 ± 13.5	111.5 ± 12.7	0.267	0.727	<.0001
DBP (mmHg)	84.1 ± 10.0	83.8 ± 10.0	84.4 ± 9.9	0.440	0.635	76.4 ± 8.9	76.5 ± 9.4	76.4 ± 8.2	0.940	0.661	<.0001
FG (mg/dl)	95.5 ± 17.8	94.7 ± 17.7	96.3 ± 17.8	0.185	0.696	91.2 ± 18.8	90.8 ± 18.0	91.5 ± 19.7	0.710	0.781	<.0001
TG (mg/dl)	130.7 ± 80.9	135.7 ± 87.5	125.4 ± 73.1	0.105	0.240	91.9 ± 56.5	94.3 ± 63.1	89.3 ± 48.6	0.361	0.280	<.0001
Cholesterol (mg/dl)	198.3 ± 35.3	198.9 ± 35.8	197.7 ± 34.8	0.690	0.611	193.5 ±34.1	192.2 ± 32.8	194.9 ± 35.4	0.426	0.988	0.027
HDL-C (mg/dl)	50.4 ± 11.1	49.9 ± 10.7	51.05 ± 11.50	0.175	0.253	60.1 ±13.9	60.8 ± 13.8	59.4 ± 14.1	0.323	0.807	<.0001
LDL-C (mg/dl)	120.8 ± 35.1	120.5 ± 36.3	121.2 ± 33.9	0.834	0.641	114.5 ± 32.7	111.6 ± 33.0	117.6 ± 32.3	0.058	0.593	0.0007
Total carotenoids (μM)	6.8 ± 8.0	6.0 ± 5.9	7.6 ± 9.7	<.0001	0.057	10.6 ± 13.6	9.4 ± 10.8	11.9 ± 16.1	0.059	0.177	<.0001
Lutein (μM)	0.8 ± 0.9	0.7 ± 0.7	0.9 ± 1.0	0.002	0.117	1.05 ±1.7	1.0 ± 1.6	1.2 ± 1.7	0.224	0.432	0.0004
Zeaxanthine (μM)	0.4 ± 0.5	0.4 ± 0.5	0.4 ± 0.5	0.424	0.828	0.4 ± 0.8	0.4 ± 0.8	0.4 ± 0.8	0.521	0.500	0.227
ß-cryptoxanthin (μM)	1.8 ± 2.6	1.7 ± 2.2	2.0 ± 2.9	0.003	0.261	2.7 ±5.4	2.4 ± 3.8	3.0 ± 6.7	0.254	0.384	0.0002
α-carotene (μM)	0.2 ± 0.3	0.2 ± 0.2	0.3 ± 0.3	0.049	0.141	0.3 ± 0.4	0.3 ± 0.3	0.4 ± 0.5	0.018	0.049	<.0001
ß-carotene (μM)	3.5 ± 4.5	3.0 ± 2.9	4.1 ± 5.7	<.0001	0.021	6.1 ± 7.1	5.4 ± 6.0	6.9 ± 8.1	0.024	0.121	<.0001

Abbreviations: SBP, systolic blood pressure; DBP, diastolic blood pressure; FG, fasting glucose; TG, triglycerides; HDL-C, high-density lipoprotein cholesterol; LDL-C, low-density lipoprotein cholesterol;
Data are presented as mean±SD.
†RFS median values are 23 in men and 21 in women.
*p-values were obtained by RFS median using Student's t-test.
**p-values were obtained by RFS median using General linear model adjustment for age, marital status, smoking, physical activity, and menopause status (women only).
‡p-values were obtained by sex using Student's t-test for continuous variables or chi-square tests for categorical variables.
